# Acute genital ulceration in a 14-month-old girl with *rhinovirus* infection and new onset B-cell acute lymphoblastic leukemia

**DOI:** 10.1016/j.jdcr.2026.03.021

**Published:** 2026-03-19

**Authors:** Mharlove Andre, Caitlin Purvis, Kiran Motaparthi

**Affiliations:** a University of Florida College of Medicine, Gainesville, Florida; b Department of Dermatology, University of Florida College of Medicine, Gainesville, Florida

**Keywords:** acute genital ulceration, Epstein-Barr virus, kissing ulcer, Lipschütz ulcer, *rhinovirus*, type III hypersensitivity

## Introduction

Acute genital ulceration (AGU), historically known as “Lipschütz ulcer,” was first described in 1912 by Benjamin Lipschütz^1^ who highlighted a series of young, nonsexually active females who presented with acute onset, painful ulcerative lesions of the vulva.^1^^,^^2^ Numerous cases of AGU have been reported, with the most common etiology being Epstein-Barr virus.^3^ The underlying infection is postulated to be transmitted via hematogenous spread of infected lymphocytes or Langerhans cell precursors or autoinoculation with saliva, urine, or cervicovaginal fluid. However, the cause is not always identified.^3^ Studies demonstrate an increased incidence in pubertal non-sexually active girls and cases that occur after the first year of menarche, with a mean age of 15.5 y.^2^^,^^4^ This report describes AGU in an infant, arising in association with *rhinovirus* infection in the setting of recently diagnosed B-cell acute lymphoblastic leukemia (B-ALL).

## Case report

**Fig 1:**
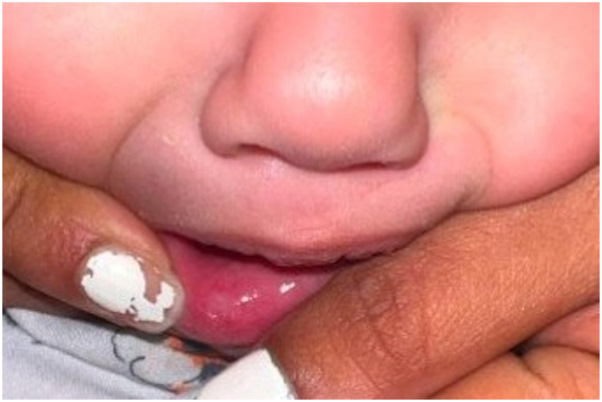
Well circumscribed ulceration with gray base and peripheral erythema on the lower mucosal lip.

**Fig 2:**
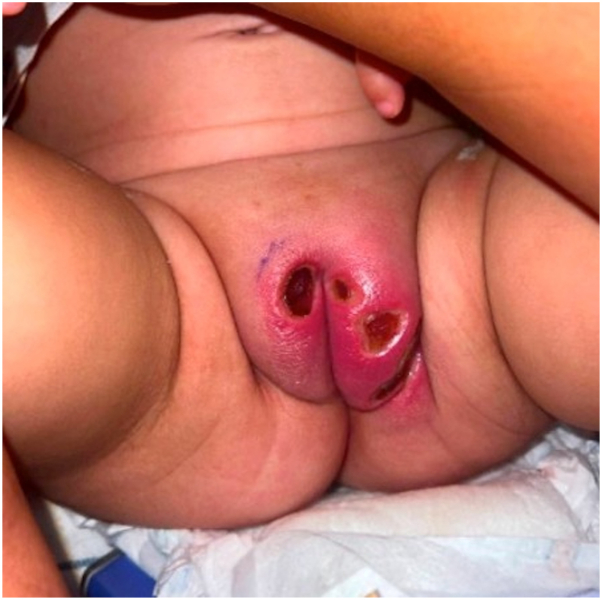
Well demarcated erythematous ulcerations with fibrinous base and peripheral edema, erythema, and induration on the labia majora.

A 14-month-old full term female with no prior past medical history who received all age-appropriate vaccinations presented to the emergency department for 1.5 weeks of intermittent fevers. Upon initial presentation, the patient was treated presumptively for streptococcal pharyngitis with a course of amoxicillin. Shortly after, the patient’s parents noticed small, dark, “pimple like” lesions developing on her vulva which rapidly progressed to deep ulcerations over 2 days, prompting the patient to return to the emergency department. Laboratory testing at that time was significant for leukopenia (2.8 × 10^3^/uL) with an absolute neutrophil count of 190 without myeloblasts and reticulocytopenia (0.2%) in the setting of anemia (hemoglobin 5 g/dL). Respiratory panel polymerase chain reaction (PCR) resulted positive for *rhinovirus*. Physical exam demonstrated a well demarcated ulcer with a light-gray base and surrounding erythema on the lower right mucosal lip (Fig 1). Large, well circumscribed bilateral ulcerations with fibrinous bases, peripheral erythema, edema, and induration were observed on the labia majora (Fig 2). No palpable inguinal lymphadenopathy was appreciated.

**Fig 3:**
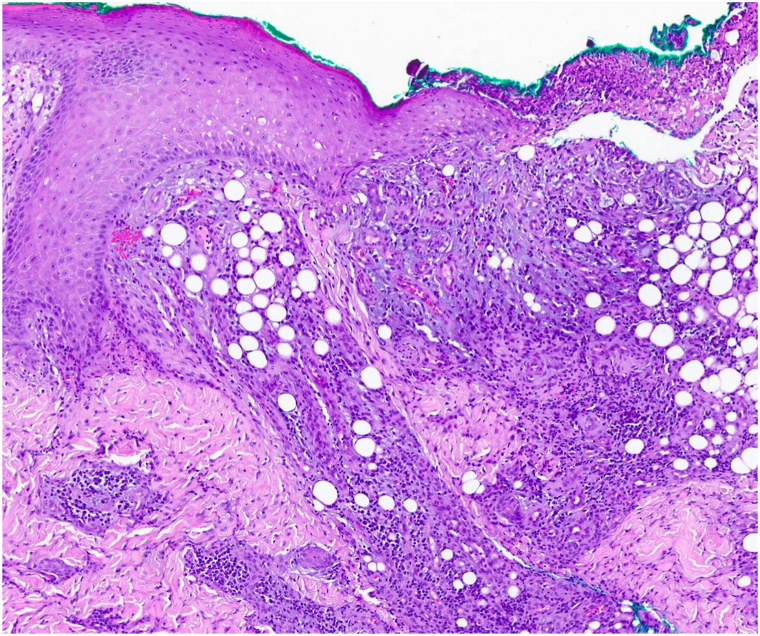
The biopsy shows an ulcer with a subjacent diffuse and perivascular mixed inflammatory cell infiltrate comprising lymphocytes, histiocytes, and neutrophils without any concomitant necrotizing vasculitic changes and without subcutaneous extension. (H&E, 100× magnification).

The differential diagnosis included AGU in the setting of *Rhinovirus* infection, ecthyma gangrenosum, and complex aphthous ulcers due to cyclic neutropenia. Further exploration of the patient’s pancytopenia with a bone marrow biopsy revealed new onset B-ALL. Blood cultures as well as PCR and serologies for Epstein-Barr virus, cytomegalovirus, human herpesvirus 6, herpes simplex virus, *parvovirus*, *influenza*, SARS-CoV-2, and *Enterovirus* were negative. Punch biopsy from an ulcer on the labium majus demonstrated mixed inflammation without vasculitis, dermal necrosis, or bacteria (Fig 3). PAS-F and GMS highlighted yeast and pseudohyphae in the stratum corneum adjacent to the ulcer, representing either colonization or superficial infection by *Candida*; Gram, Fite-Faraco, and Zeihl-Neelsen stains were all negative. Tissue cultures were negative for bacteria and fungi other than *Candida*. Based on the patient’s presentation, infectious workup, and biopsy result, she was diagnosed with AGU in the setting of *Rhinovirus* infection and newly diagnosed B-ALL. Treatment of her B-ALL included intrathecal chemotherapy and three-drug induction therapy with vincristine, methotrexate, and cytarabine. The vulvar lesions healed over 22 days with mupirocin and barrier creams. Her oral ulcer also healed.

## Discussion

AGU commonly presents with the rapid onset of large (>1 cm) ulcerations with red-violaceous borders, a necrotic base, and painful “kissing lesions” typically localized to the vulva, most commonly in adolescent females. While concurrent oral and vulvar ulcers are more commonly observed in complex aphthosis, oral ulcers clinically resembling aphthous ulcers commonly occur in patients with acute genital ulceration. Reported causes of AGU are included in Table I. This patient was found to have a *rhinovirus* infection, which is uncommonly associated with AGU. Pancytopenia is also not typically seen in AGU; however, this patient’s concurrent B-ALL most likely predisposed her to viral infection and subsequent AGU.

**Table I:** Reported causes of acute genital ulceration

Category	Specific cause	References
Infectious (nonsexually transmitted)		
	*Epstein Barr virus*	2, 3, 4, 5
	*Cytomegalovirus*	2,4,5
	*Influenza B*	2,5
	*Salmonella* paratyphi	2,5
	*Mycoplasma* species	4,5
	*Toxoplasma gondii*	4,5
	*Mumps virus*	5
	*Adenovirus*	5
	*Parvovirus B19*	5
	*SARS-CoV-2*	6
Systemic disorders		
	IgA deficiency	5
Drugs		
	Nonsteroidal anti-inflammatory drugs	2
Other		
	Idiopathic	2
	COVID-19 postvaccination	6

*IgA*, Immunoglobulin A.

For the differential diagnosis, ecthyma gangrenosum was considered; however, this was excluded as skin biopsy did not show characteristic pauci-inflammatory septic vasculitis with gram negative bacilli and resultant transmural necrosis and intravascular thrombosis. Tissue culture also failed to identify gram negative bacteria. Cyclic neutropenia was also considered, but this condition typically presents with recurrent fevers and oral aphthous ulcers, not genital ulcers.^7^ This patient’s parents did not endorse a history of prior neutropenia or recurrent infections, the ulcers were predominantly located on the vulva rather than oral mucosa, and an underlying cause of neutropenia (leukemia) was ultimately identified. Finally, viral infections including herpes simplex virus and cytomegalovirus infection were considered but excluded based on histopathology, PCR, and serology.

The exact pathogenesis of AGU is not well understood.^8^ One hypothesis suggests a type III hypersensitivity reaction caused by viral infection leading to immune complex deposition, activation of complement, microthrombosis, and eventually tissue necrosis.^3^ Cytolysis secondary to viral replication in vulvar keratinocytes and inflammatory response to the release of viral antigens have also been suggested.^3^ The classic histologic findings are those of a mixed inflammatory cell infiltrate comprising lymphocytes, histiocytes and neutrophils with a small vessel neutrophil enriched vasculitis. In this case, the lack of vasculitic changes could reflect a deficient B-cell immune response in the setting of B-ALL. Commonly noted treatment modalities for AGU include sitz baths, topical analgesics, nonsteroidal anti-inflammatories, acetaminophen, topical steroids, and systemic steroids.^6^

AGU has been diagnosed previously in patients with leukemia, most notably acute myeloid leukemia. One case describes a 16-year-old girl who initially presented with fever and a 2-m history of a nontender, superficial, ulcerative lesions with a necrotic base over the labium majus.^9^ Testing significant for thrombocytopenia prompted bone marrow aspiration later revealing an increased number of myeloperoxidase positive myeloblasts, confirming acute myeloid leukemia. A similar case was reported in a 22-year-old woman whose initial presentation of AGU and acute myeloid leukemia included genital ulcers, ulcerations of the oral mucosa, and pustules of the inguinal and axillary regions.^10^ Among younger children, AGU due to Epstein-Barr virus or without identified infectious etiology has been described in girls as young as 11 months of age.^4^^,^^8^ There are no prior published cases describing AGU in association with underlying *rhinovirus* infection. PCR testing for common respiratory viruses may support the workup of AGU.

AGU is a diagnosis of exclusion that requires a thorough workup and consideration of age, history, clinical symptoms, prodrome, time course of ulcerative lesion onset, and location of ulcerations. Treatment is supportive, and healing typically occurs within 2 to 6 weeks.

## Conflicts of interest

None disclosed.
